# Sugars and Polyols of Natural Origin as Carriers for Solubility and Dissolution Enhancement

**DOI:** 10.3390/pharmaceutics15112557

**Published:** 2023-10-30

**Authors:** Madan Sai Poka, Marnus Milne, Anita Wessels, Marique Aucamp

**Affiliations:** 1 Department of Pharmaceutical Sciences, School of Pharmacy, Sefako Makgatho Health Sciences University, Molotlegi Street, Pretoria 0208, South Africa; marnus.milne@smu.ac.za; 2 Centre of Excellence for Pharmaceutical Sciences (Pharmacen), North-West University, Hoffman Street, Potchefstroom 2520, South Africa; anita.wessels@nwu.ac.za; 3 School of Pharmacy, University of the Western Cape, Robert Sobukwe Drive, Cape Town 7130, South Africa

**Keywords:** sugars, polyols, solubility, dissolution, solid dispersions

## Abstract

Crystalline carriers such as dextrose, sucrose, galactose, mannitol, sorbitol, and isomalt have been reported to increase the solubility, and dissolution rates of poorly soluble drugs when employed as carriers in solid dispersions (SDs). However, synthetic polymers dominate the preparation of drugs: excipient SDs have been created in recent years, but these polymer-based SDs exhibit the major drawback of recrystallisation upon storage. Also, the use of high-molecular-weight polymers with increased chain lengths brings forth problems such as increased viscosity and unnecessary bulkiness in the resulting dosage form. An ideal SD carrier should be hydrophilic, non-hygroscopic, have high hydrogen-bonding propensity, have a high glass transition temperature (*T_g_*), and be safe to use. This review discusses sugars and polyols as suitable carriers for SDs, as they possess several ideal characteristics. Recently, the use of low-molecular-weight excipients has gained much interest in developing SDs. However, there are limited options available for safe, low molecular excipients, which opens the door again for sugars and polyols. The major points of this review focus on the successes and failures of employing sugars and polyols in the preparation of SDs in the past, recent advances, and potential future applications for the solubility enhancement of poorly water-soluble drugs.

## 1. Introduction

Poor aqueous solubility is a major concern for new chemical entities (NCEs) and many of the existing active pharmaceutical ingredients (APIs) developed for oral drug delivery, where these substances have great pharmacological and permeability potential. An increasing proportion of potential new drug candidates (40–70%) are classified as Biopharmaceutical Classification System (BCS) Class II or IV drugs, and the formulation and commercialisation of these as oral dosage forms presents great challenges as they show sub-optimal dissolution; absorption; and, hence, bioavailability [1]. The poor aqueous solubility has a negative effect on dissolution, resulting in the incomplete absorption of these APIs in the gastrointestinal tract; hence, the bioavailability is affected detrimentally [2,3]. Several techniques are used to overcome this problem, which include physical and chemical modifications of the drug through micronization, crystal engineering, salt formation, the addition of surfactants, solid dispersions with hydrophilic carriers, complexation, hydrotropy, eutectic mixtures, and amorphous systems, to mention but just a few [2,4]. Among these solid-state alterations, solid dispersions and co-amorphous and co-crystal systems have taken the lead over the past two decades. One of the common factors among these techniques that plays a significant role in solubility and bioavailability enhancement is the type of carrier, co-former, or excipient used [5,6]. In the selection of a carrier, preference is given to carriers that are hydrophilic in nature [5], have high hydrogen bonding propensity, have higher glass transition temperatures (*T_g_*), are non-hygroscopic [7], and form part of the United States Food and Drug Administration (FDA)-approved Generally Recognized as Safe (GRAS) list [8].

Naturally occurring sugars of several types, such as monosaccharides (glucose, fructose, galactose); disaccharides (sucrose, maltose, lactose); and sugar alcohols (polyols) such as sorbitol, mannitol, xylitol, erythritol, maltitol, lactitol, and isomalt, have been reported to enhance the solubility of drugs. The FDA designates sugars and polyols as GRAS excipients [9,10], and in addition to their proven safety, these compounds exhibit good water solubility [11] and possess hydroxyl groups (-OH) attached to each carbon atom in the molecule, which, in theory, provides hydrogen-bonding sites for drug molecules, thereby already meeting three of the abovementioned criteria in terms of suitable solid-state co-formers.

There are several ways in which sugars and polyols have been used to enhance the solubility of drugs. One predominant method used is to form solid dispersions of the drug [12]; another approach is the formulation of eutectic mixtures [13,14]. Polyols such as xylitol [15,16], mannitol [15], and sorbitol [17] have been reported as potential co-formers for developing co-crystalline systems. However, efforts made in exploring sugars and polyols in the making of co-crystals and co-amorphous systems are few to none. The low molecular weight, safety, good glass-forming ability, and (in some instances) high *T_g_* of sugars [18] make them an interesting option to explore as excipients for drug–excipient co-amorphous and co-crystalline systems. The current review focuses on summarising the use of naturally occurring sugars and polyols for the enhancement of solubility and the dissolution rate of poorly soluble drugs. Furthermore, it seeks to understand the pros and cons in relation to the various techniques used; to provide a theoretical basis for their application in the formulation of co-amorphous and co-crystalline systems; and finally, to identify potential opportunities for future research.

## 2. Solid Dispersions

**Figure 1:**
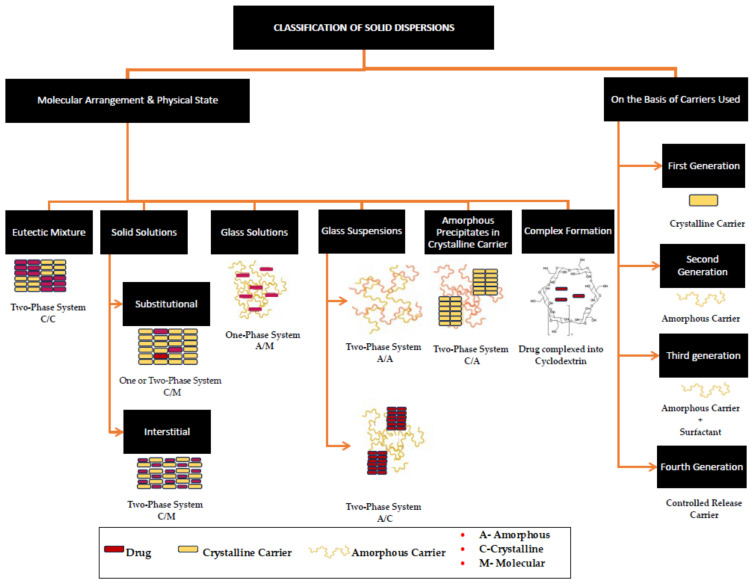
A schematic depiction of the classification of solid dispersions, obtained and reproduced with changes from Ref. [25] and the Association of Pharmaceutical Teachers of India in accordance with Creative Commons Attribution—Non-Commercial 4.0 International (CC BY-NC 4.0).

Since first introduced by Sekiguchi and Obi in 1961, solid dispersions (SDs) have been the most predominantly used solid-state modification technique, found to have significant commercial success [19,20,21]. Since the first FDA-approved SD, Cesamet^®^ in 1985 (Bausch Health Companies Inc., Laval, QB, Canada), over 30 amorphous solid dispersions of Class II and Class IV drugs are now currently marketed as oral dosage forms, signifying the importance of this technique [20,21,22]. A solid dispersion (SD) can be defined as a solid-state mixture of one or more hydrophobic drugs and one or more hydrophilic carriers. The process involves the distribution of the drug molecules in the hydrophilic carrier matrix at the molecular or colloidal level via melting (fusion), quench cooling, solvent evaporation, spray drying, freeze drying, or hot-melt extrusion [5,23]. In SDs, either the particle size of the drug is reduced, resulting in the increased surface area of the drug upon contact with the solvent, or a crystalline pure drug is converted into an amorphous form leading to increased solubility [23]. Based on the miscibility of the drug in the carrier, the final solid-state form (crystalline or amorphous), and the molecular arrangement, SDs can be classified as (a) simple eutectic mixtures, (b) solid solutions, (c) glass solutions, (d) glass suspensions, or (e) amorphous precipitations in a crystalline carrier [5,23,24]. A summary of different types of SDs and their solid states is illustrated in Figure 1.

SDs carry common properties in the sense that the carriers used are typically hydrophilic and inert and exhibit the common principles of solubility and dissolution enhancement. The main and common mechanisms of solubility enhancement are a reduction in the particle size of the crystallite API to its molecular size for molecular dispersion or ultrafine particulates in the making of solid suspension. This increase in surface area results in the enhanced wettability and dispersibility of the drug into the dissolution medium [5,26]. According to the BCS, Class II drugs exhibit a dissolution rate of limited absorption, and therefore, solid dispersion technology is a promising approach to enhancing dissolution. While the commercial success of BCS Class II drugs is limited by their inherent low dissolution, Class IV drugs show permeation-limited absorption. Among the various physical and chemical modification techniques available, SDs provide a wide range of opportunities, as they offer greater flexibility in formulating oral drug delivery systems given the availability of various processing and excipient alternatives [19]. The ease of preparation and regulatory compliance requirements of SDs in comparison with techniques such as those involving the formation of solvates, hydrates, salt formation, the inclusion of polar or ionizable groups, and co-crystallisation makes them a more favourable approach [22,27,28].

SDs can be prepared by applying a wide range of techniques, including simple fusion; hot-melt extrusion that uses heating; solvent-based preparation techniques such as simple kneading with selected solvents; solvent evaporation; spray-drying; freeze-drying; and solvent-free methods such as milling with a ball-mill or air-jet milling. Though the preparation of SDs is relatively simple, they are accompanied by unique challenges such as the thermal instability of their APIs and carriers; a lack of solubility in the hydrophilic carriers in organic solvents, leading to phase separation during the process; high costs; low yields associated with techniques such as spray-drying; and often, scalability for commercial purposes. A summary of various techniques used in the preparation of SDs, their advantages and limitations are discussed in the following table (Table 1).

### 2.1. Solid Dispersions of Sugar Carriers

A variety of hydrophilic carriers have been reported in the formulation of SDs, wherein crystalline carriers such as sugars have been studied in the formulation of first-generation Class C-C (crystalline drug dispersed in crystalline carrier) SDs [23,34]. 

Allen et al. [35] first reported the use of sugars in the preparation of SDs for the solubility enhancement of several corticosteroids. Sugar-based glass dispersions were prepared using the fusion method, where the sugars (dextrose, galactose, and sucrose) formed an amorphous environment for the dispersion of the drug molecules when rapidly cooled from a molten state. The authors reported a good glass-forming ability and an increased dissolution rate for the drug for all the sugars used. Dissolution studies indicated a bi-phasic drug release with rapid initial drug release followed by prolonged drug release. This phenomenon was attributed to the formation of partial glass solutions in the sugars, as they were not heated to the melting point to avoid the degradation of the corticosteroids. This resulted in the partial distribution of the drug at the molecular level. Despite this, SDs were found to increase the dissolution rate of the drugs in comparison with the pure, unaltered drug. The improved dissolution rate was due to a reduction in the particle size of the drug to a very fine state (Phase—I) and increased wettability (Phase—II). The study also reported some challenges such as discolouration, especially observed for sucrose and dextrose, indicating the degradation of the sugars and the hygroscopic nature of the resulting SDs. 

To overcome these challenges, Allen et al. [36] prepared ternary sugar-based SD systems of corticosteroids with sucrose–mannitol (1:1) and sorbitol–mannitol (1:1) in a drug-to-carrier ratio of 1:19. The results were indicative of the good miscibility of the drugs in the sugar carrier systems, less hygroscopicity, and no discolouration. This was mainly attributable to the presence of mannitol in the mixtures, as the binary solid dispersion of mannitol as a sugar carrier formed a stable, non-hygroscopic, free-flowing dispersion system that revealed no discolouration during melting and good miscibility with the drugs. Interestingly, the lack of discolouration in the sucrose–mannitol systems was due to a reduction in the melting point of the system, indicating the formation of a eutectic mixture. Tablets prepared from these solid dispersions showed a significant increase in the dissolution rate over the tablets with the drug alone (30% vs. 80% drug release in 10 min). This was ascribed to the enhanced wettability of the drug particles facilitated by the hydrophilic sugar carriers.

Ghanem et al. [37] prepared SDs made of sulfamethoxazole via the quench-cooling technique using non-reducing sugars (glucose and galactose) and reducing sugars (maltose and sucrose). SDs consisting of a drug-to-sugar ratio of 1:1 were prepared by heating the mixture slightly above the melting point of the individual sugars to avoid caramelisation, which resulted in a glassy material with sulfamethoxazole dispersed as solid particles. In comparison with the drug, which showed only approximately 20% drug dissolution, the SDs consisting of glucose and maltose showed 100% drug dissolution within 5 min, while the galactose-based SDs showed complete drug dissolution only after 90 min. Similar results were reported in earlier studies [35], where drug releases from galactose-based SDs were low in comparison with other sugars and at times lower than the drug itself. Similar to previous studies, the increase in the solubility of the solid dispersions was attributable to the wettability of the drug particles. 

The authors further reported forming a complex between the free carbonyl group of the non-reducing sugars and the amino group of the drug. This complexation could have potentially resulted in a new solid-sate form of the APIs, which is worth further investigation using advanced characterisation techniques such as differential scanning calorimetry (DSC) and powder X-ray diffraction (PXRD). It was also reported that the complexation increased with an increase in the weight ratio of the sugar, as high as 1:50 for full complexation in the case of glucose. The study also reported that the observed complexation only occurred after using the fusion technique, whereas solvent evaporation from a drug:sugar mixture dissolved in an organic solvent, such as methanol, did not result in drug:sugar complexation. This is owing to the poor solubility of sugars in organic solvents [38] and the fact that the use of a common solvent does not necessarily have a positive effect on the chemical interaction between a drug and a carrier [8].

SDs of carbamazepine (CBZ) and nitrazepam were prepared using lactose and galactose via the fusion method at room temperature. The resulting sugar-based SD-system, consisting of a CBZ-to-carrier ratio of 1:3, showed an enhanced dissolution rate with a bi-phasic drug release profile, similar to the studies discussed above. In another study, SDs of CBZ were prepared via a quench-cooling process using lactose as the carrier. The results indicated an increase in the dissolution rate with an increase in the carrier concentration. The enhanced dissolution was mainly attributable to the reduced particle size and increased wettability of the drug, increasing the chances of hydrogen bond formation between the hydroxyl group of lactose and the carboxylic acid group of the CBZ [39]. In addition to the findings mentioned above, the authors also noted that the dissolution rates of the SDs were more consistent than those of the physical mixtures and coprecipitates. Both these studies confirm the partial amorphization of the drug, which is the main reason for an enhanced dissolution rate. 

In preparing ibuprofen SDs via the fusion method, sugar carriers (icing sugar (sucrose), dextrose, mannitol, and lactose) have been used and compared against the dissolution rate of the pure drug. In vitro dissolution studies have reported the effect of sugar concentration (drug-to-sugar ratios of 4:1, 2:1, 1:1, and 1:4) on drug release. The increase in the sugar concentration increased the drug release. However, none of the sugar-based SDs have reported 100% drug release after 60 min and, therefore, an increase in the sugar content had an insignificant effect [40].

Similar results were reported by Madgulkar et al. [7] when SDs of clotrimazole were prepared using sugars (D-fructose, D-dextrose, and D-maltose). The results indicated a decrease or insignificant change in the saturation solubility and dissolution rate for dextrose, lactose, and maltose, while the opposite was the case for fructose. This phenomenon in sugars was explained by Etman and Naggar [41] as competition for hydrogen bonding with water molecules, which facilitates the solvation of the drug. An increase in sugar concentrations results in having many hydrogen-bonding sites competing against the polar groups of the drug molecules. Another proposed theory indicates that an increase in sugar concentration results in supersaturation, leading to the crystallisation of the sugar and growth in the crystal size [7]. 

One of the important characteristics of carriers used in the preparation of SDs is their stability in heat [5]. In the studies described above, it was found that the application of sugars in SDs shows limitations based on the occurrence of the Maillard reaction when they are heated to above their melting point, causing the degradation of the sugar. Often, the solid dispersions cannot be heated to melt both the drug and the carrier, resulting in the partial distribution of the drug at the molecular level; hence, a bi-phasic drug release is observed in most cases, and the drug cannot be released rapidly. 

In addressing the problem of miscibility at the molecular level, ultrasonication was applied after melting an indomethacin–glucose mix and prior to cooling it to room temperature. This assisted in dispersing the drug and sugar homogeneously, resulting in the formation of two-phase amorphous–amorphous solid dispersions (Class A-A), signified by an amorphous drug dispersed in an amorphous carrier. While dissolution studies showed an eight-fold increase in drug release, pharmacokinetic studies only showed an increase in bioavailability of 1.9-fold. It is worth noting that the resultant SDs remained amorphous for 2 years at room temperature [42]. Of note, ultrasonication might not be the sole reason for the formation of ASDs, as the glass-forming ability of both the drug and sugar plays a significant role [22]. 

While the fusion and quench-cooling methods have been extensively utilized, they are mostly conducive to forming solid suspensions rather than solid solutions. This inherent limitation constrains the potential of sugars as carriers to significantly enhance drug solubility and dissolution rates. An analysis of the studies reviewed (Table 2) indicates that factors such as a low degradation threshold, a lack of miscibility between the drug and sugar at elevated temperatures, hygroscopicity, and solution-mediated phase transitions represent the principal shortcomings of sugars in SD preparation via the fusion or quench-cooling methods. The recrystallisation of the drug during dissolution can predominantly be attributed to the rapid dissolution of sugar in the solvent due to its high solubility. This leads to the creation of a supersaturated environment and competes for hydrogen-bonding sites with the solvent. 

In the preparation of SDs, solvent evaporation is also considered one of the most commonly used methods, particularly when dealing with drug–carrier mixtures where one of the compounds either possesses a high melting point or undergoes degradation when heated [43]. Etoricoxib SDs were prepared with sugar carriers such as lactose and sucrose in 1:1 and 1:5 ratios using the solvent evaporation technique. The drug–sugar mixture is dissolved in ethanol and subsequently heated in a water bath at 60 °C to facilitate solvent removal. An FTIR analysis of these SDs indicated potential intermolecular hydrogen bonding between the S=O group of etoricoxib and the O–H group of the sugar carriers. X-ray diffraction (XRD) and differential scanning calorimetry (DSC) unveiled the absence of characteristic peaks associated with the drug, coupled with the reduced enthalpy of etoricoxib, indicating a shift towards an amorphous state for etoricoxib within the SDs. Notably, unlike the melting or fusion method, the SD systems formed here were Class A-C, denoting the presence of an amorphous drug dispersed in a crystalline carrier matrix. Equilibrium solubility studies indicated an increase in solubility by 1.8- and 1.5-fold for SDs consisting of lactose and sucrose, respectively, in a 1:5 ratio [44].

The hydrochloride salt of amino sugar, glucosamine hydrochloride (G-HCL), was studied for its potential as a hydrophilic carrier in the preparation of SDs for poorly soluble drugs. SDs of CBZ [45] and acyclovir (ACV) [46] were prepared using the solvent evaporation method. In the case of CBZ, SDs were prepared using single (ethanol or acetone) and binary solvent systems mixed with water. The study evaluated the effect of the carrier concentration and the solvent system on CBZ solubility and the dissolution rate. Interestingly, the results indicated that an increase in the carrier concentration did not consistently result in increased dissolution rates when a single solvent system was used. This phenomenon was mainly attributed to the limited solubility of G-HCL within the organic solvents. To corroborate this finding, dissolution profiles of SDs prepared with a binary solvent system (ethanol–water or acetone–water) were investigated. Notably, SDs from the binary solvent systems showed increased dissolution rates with increasing carrier concentration, underscoring the important role the solvent system plays in facilitating the dissolution of both the drug and the carrier. The same trend was also observed for the solubility study results. 

SDs of ACV were prepared using ethanol, wherein the drug and carrier were mixed until the solvent completely evaporated. FTIR results indicated that ACV SDs showed potential intermolecular interactions between the N-H group of ACV and the O-H group of G-HCL. Despite the presence of an amine group in CBZ, no intermolecular interactions were reported in the previous study. The difference may be attributed to the use of grinding in the presence of a solvent, which causes the loosening of the molecules at the reaction sites, leading to a chemical change or phase transition [47]. Furthermore, the grinding process also facilitated the reduced particle size of the ACV, resulting in partial amorphization and enhanced aqueous solubility. Comparatively, ACV physical mixtures and SDs showed a significant increase in solubility of 6- and 12-fold, respectively, in contrast with the pure drug. The study also indicated that the increase in the concentration of the carrier only resulted in a mild-to-moderate increase in aqueous solubility, aligning with findings from the various studies mentioned earlier. 

In comparison with the fusion method, SDs prepared via solvent evaporation often show evidence of intermolecular interactions, which could explain the absence of the “parachute” effect during dissolution studies. Intermolecular interactions can suppress molecular mobility, thereby inhibiting the formation of the crystal nucleus and recrystallisation [48]. Nevertheless, several challenges were reported, including the limited solubility of sugars in organic solvents and the necessity of removing organic solvents to avoid solvation during storage. Strategies such as employing binary solvent systems yielded positive results to a certain extent, based on the concentration of the sugar. Further studies could delve into the use of binary solvent systems with less polarity difference and the addition of surfactants to enhance the solubility of sugars in organic solvents.

Freeze-drying stands as one of the most widely used techniques for the preparation of amorphous solid dispersions (ASDs) [49]. In addressing the limitations associated with the fusion and solvent evaporation methods, several authors have explored an alternative approach involving SDs with sugar carriers. In two different studies, van Drooge et al. [50,51] investigated the incorporation of lipophilic drugs into sugar glasses via the lyophilization process, employing a solvent mixture consisting of water and tertiary butyl alcohol. Sugar-glass-based ASDs made of diazepam [50], nifedipine, Δ^9^-tetrahydrocannabinol (THC), and cyclosporine A [51] were successfully prepared using trehalose, sucrose, and two inulins (inulinDP11 and inulinDP23). Firstly, the results of the study underscore the effectiveness of the lyophilization process in transforming lipophilic drugs into amorphous forms, incorporating such drugs into sugar glasses. The dissolution behaviour studied for tablets containing diazepam SDs exhibited anomalous dissolution, characterized by nonlinear and unpredictable drug release patterns in the case of trehalose, sucrose, and inulinDP11. This phenomenon primarily stems from the high aqueous solubility of sugars, leading to the rapid release of the drug and the formation of a supersaturated system. This resulted in diazepam undergoing solution-mediated phase transformation and recrystallisation [50]. 

This phenomenon was scrutinized by Srinarong et al. [52], who found similar results, wherein the drug loading and solubility of the carrier were identified as pivotal factors in recrystallisation prevention. Notably, the high *T_g_* exhibited by inulins conferred enhanced physical stability compared with trehalose and sucrose. It is worth noting that the conversion of crystalline drugs into their amorphous counterparts is dependent on the drug. This parameter varies from one drug to another, based on the closeness of the *T_g_* to the melting point [51]. 

Palatinose, α-maltose, and trehalose were employed in the development of completely amorphous sugar-based solid dispersions (SAS-SDs). These systems were prepared by initially subjecting the sugars to freeze-drying, followed by their solubilization in an organic solvent containing the hydrophobic drug. The initial freeze-drying step led to the formation of amorphous sugars, thereby enhancing their solubility in organic solvents. This technique exhibits great promise, as it allows for the solubilization of substantial quantities of sugars, up to 100 mg/mL, in methanol [53]. Subsequently, the resultant mixture is further processed via vacuum-drying [54,55,56] to remove the organic solvent. The resultant material showed no discernible endothermic events during DSC analysis, indicating the conversion of both components into an amorphous form, thereby yielding ASDs. This noteworthy phenomenon was observed for SDs containing drug loadings ranging between 1 to 10% *w*/*w*, where a small endothermic event signified the incomplete amorphization of the drug, thus emphasizing the influence of drug loading [55]. 

SDs investigated in all of the studies [54,55,56] showed a *T_g_* below 40 °C, indicating the potential vulnerability of these systems to instability during storage and solution-mediated phase transitions during the drug dissolution process. This phenomenon was unequivocally substantiated with dissolution studies, where a distinctive “spring and parachute” effect was consistently observed across these investigations. Furthermore, it was noted that the *T_g_* of these systems decreased with an increase in the drug content, attributable to the plasticizing effect of the hydrophobic drug [56]. The decrease in the *T_g_* of the SDs directly relates to the *T_g_* of the pure sugars [55,56]. However, when the SAS-SDs are subjected to thermal treatments above the melting point of the drug followed by rapid cooling (quenching), a distinctive plateau emerges in the subsequent drug dissolution profile [56]. 

To address the issue of low *T_g_*, Takeda et al. [57] devised a method involving the heat treatment of ASDs formed through the freeze-drying process. Specifically, SDs comprising α-maltose and hydrophobic drugs were prepared via freeze-drying and subjected to heat treatments between 30–120 °C for durations extending up to 120 min. The results of the study indicated that the *T_g_* of the SDs increased with an increase in temperature and the time of heating, thereby showing the direct relationship of these preparation parameters with the SD’s *T_g_*. Moreover, the dissolution studies showed a marked increase in drug release from the heat-treated ASDs in comparison with the untreated ASDs. However, both the SDs showed the typical “spring and parachute” dissolution profiles. It is worth noting that the authors did not report on the stability of the heat-treated SDs, which represents an intriguing avenue for future exploration, especially concerning the change in *T_g_* during storage.

Various alternative methods, such as kneading, centrifugal spinning, and milling, have been reported for the preparation of SDs using sugars. In the case of allopurinol SDs, the kneading method was employed using maltose, sucrose [58], lactose, and mannitol [59] as sugar carriers. In all cases, water was used as a solvent to wet the drug-carrier mixtures (1:1, 1:3, and 1:5), followed by kneading for 45 min, and they were dried and sieved to achieve a powder with an average particle size of 420 µm. The authors noted a significant improvement in drug release during the dissolution studies. However, the effect was primarily observed for the first two intervals (5 and 10 min), while from 15–60 min, the drug release between the SDs and the pure drug did not differ much for any of the ratios. Fourier-transform infrared (FTIR) spectroscopy results showed no interaction between the drug and the sugar, which could be attributed to the low aqueous solubility of allopurinol. In a separate study, Dai et al. [60] prepared co-crystals of allopurinol using liquid-assisted grinding (LAG), where methanol and isopropyl alcohol were used as solvents. As the solubility of drugs and co-formers in common solvents play a pivotal role in intermolecular interactions [8], the use of a single or binary solvent system that can provide moderate-to-high solubility for both the drug and sugar would be interesting to explore.

Saito et al. [61] used the roller compaction method to prepare griseofulvin (GF) SDs with lactose and maltose as carriers. A mixture of GF and sugar carrier at a weight ratio of 1:4 resulted in a markedly reduced particle size after passing through the roller mill for 20 min. The formation of an amorphous mixture was confirmed by the absence of characteristic diffraction peaks during X-ray diffraction analysis. Dissolution studies indicated a 35- to 40-fold increase in drug release for both sugar-based ASDs. This study, however, reported processing problems stemming from the heavy sticking of the sugars to the rollers, an aspect that may be reduced with the use of the ball milling technique. Additionally, the SDs converted into a crystalline state within 24 h when exposed to accelerated storage conditions, indicating instability.

Sucrose-loaded micro-fibrous SDs of olanzapine and piroxicam were prepared using the centrifugal spinning method. The characterization of the microfibers via thermal analysis and hot-stage microscopy confirmed the formation of ASDs by both drugs. Subsequent spectroscopic analysis indicated possible intermolecular interactions between the hydroxyl groups of sucrose and the proton-accepting groups of olanzapine. Dissolution studies, under non-sink conditions, demonstrated rapid drug release, resulting in a three-fold increase in olanzapine and a 1.7-fold increase in piroxicam. The *T_g_* values of these systems were reported to be much higher (about 70 °C), indicating the possible reason for a lack of recrystallisation during dissolution. The study reported achieving high production yield (85%) and drug-loading efficacy (90%) using this method [62]. 

Despite the several drawbacks reported, recent studies [57,62] have highlighted the potential of using sugars as carriers for SDs to improve the dissolution and solubility of poorly water-soluble drugs. However, further research may be needed to optimize the formulation conditions and assess the long-term stability and practical applicability of sugar-based SD formulations for drug delivery.

**Table 2:** Summary of SDs reported in the literature obtained by using various sugars as carriers.

Method	Sugar	Drug: Sugar (*w*/*w*)	Drug	Solubility	Dissolution	Remarks	Ref.
Fusion	Dextrose Fructose Maltose	1:3, 1:1, and 3:1	Clotrimazole	Fructose showed a slight increase at a 1:1 ratio	Increased dissolution rate for fructose SDs. Increased with an increase in sugar concentration.	Partial dispersion of drug at molecular level.	[7]
	Dextrose Galactose Sucrose	1:33 and 1:40	Corticosteroids	N/A	Increased dissolution rate with bi-phasic drug release.	Partial dispersion of drug at molecular level. Hygroscopic and heating resulted in discolouration.	[35]
	Sucrose–mannitol (1:1) Sorbitol–mannitol (1:1)	1:19	Corticosteroids	N/A	Increased dissolution rate with bi-phasic drug release. Galactose showed smaller dissolution rate.	Sucrose-mannitol eutectic showed less hygroscopicity and no discolouration.	[36]
	Lactose Galactose	1:3	Carbamazepine Nitrazepam	N/A	Increased dissolution rate with bi-phasic drug release. Galactose showed slower dissolution rate.	Partial dispersion of drug at molecular level.	[63]
	Dextrose Icing Sugar Lactose	4:1, 2:1, 1:1, and 1:4	Ibuprofen	N/A	Only 80% of the drug was released within 60 min.	An increase in the sugar concentration had an insignificant effect on drug release.	[40]
Quench Cooling	Glucose Galactose Maltose Sucrose	1:1	Sulfamethoxazole	Sugars with free carbonyl group showed slight increase in solubility.	A 100% drug release in 5 min (glucose and maltose). Galactose showed slower dissolution rate.	Partial dispersion of drug at molecular level. Hygroscopic and heating resulted in discolouration.	[37]
	Lactose	1:1, 1:3, 1:5, and 1:10	Carbamazepine Ethenzamide	N/A	Increase in dissolution rate with increase in carrier concentration. Five- to eight-fold increase in dissolution rate.	Hydrogen bonding with amide and carboxyl groups of the CBZ.	[39]
	Glucose	1:0.03	Indomethacin	N/A	Eight-fold increase in dissolution rate.	Ultrasonication of the melt increased the miscibility between the drug and carrier.	[42]
Solvent evaporation	Lactose Sucrose	1:1 and 1:5	Etoricoxib	1.5 to 1.8-fold at 1:5 ratio	N/A	Intermolecular hydrogen bonding between S=O group of etoricoxib and O–H group of sugar carriers.	[44]
	G-HCL	4:1, 2:1,1:1, 1:2, and 1:4	Carbamazepine	Solubility of solid dispersions is lower than pure drug and physical mixtures	Concentration of carrier and solvent system used affected the dissolution rate.	Presence of water in the binary solvent system reduced the dissolution rate because of the formation of the dihydrate form of the CBZ.	[45]
	G-HCL	1:1, 1:2, 1:3, 1:4, and 1:5	Acyclovir	12-fold increase	Higher concentrations of carriers (1:4 and 1:5) showed reduced dissolution due to reduced access of the ACV to dissolution medium.	Decreased dissolution rate during storage. Hydrogen bond between amine group of ACV and O-H of G-HCL.	[46]
Freeze drying	Trehalose, Sucrose, InulinDP11 InulinDP23		Diazepam Nifedipine THC Cyclosporine A	N/A	Solution-mediated phase transition in the case of trehalose, sucrose, and inulinDP11 SDs.	The chain length of inulin affected the *T_g_* of SDs, dissolution behaviour, and stability.	[50,51]
Freeze drying followed by vacuum drying	Sucrose α-maltose Trehalose α-lactose	1:10	Fat-soluble flavours	N/A	A 100-times increase for α-maltose, trehalose and maltitol in methanol.	ASDs exhibited solution-mediated phase transition after 200 s.	[54]
	Trehalose α-maltose Palatinose	1 to 10% *w*/*w*	Indomethacin, Ibuprofen, Gliclazide, Nifedipine	20–1000% increase	Palatinose and/or α-maltose showed superior dissolution.	ASDs exhibited solution-mediated phase transition because of low *T_g_.*	[55]
	Trehalose α-maltose Palatinose	0.1 to 10% *w*/*w*	Curcumin	N/A	α-maltose and trehalose showed superior dissolution.	ASDs exhibited solution-mediated phase transition.	[56]
Kneading	Lactose Maltose Sucrose	1:1, 1:3, and 1:5	Allopurinol	N/A	No significant increase in dissolution rate.	No intermolecular interactions found. Slight increase in dissolution rate due to partial amorphization.	[58,59]
Roller compaction	Lactose Maltose	1:4	Griseofulvin	N/A	A 35- to 40-fold increase.	Processing problems due to sticking and physical instability.	[61]
Centrifugal spinning	Sucrose	1:9	Olanzapine Piroxicam	Increase in solubility is proportional to sucrose concentration	A 3- and 1.7-fold increase in dissolution rate of olanzapine and piroxicam, respectively.	Intermolecular interactions observed with olanzapine SDs. No solution-mediated phase transition was observed after 4 h of dissolution	[62]

### 2.2. Solid Dispersions of Sugar Alcohol (Polyol) Carriers

Sugar alcohols, also known as polyols, encompass both monosaccharides (erythritol, xylitol, sorbitol, mannitol) and disaccharides (lactitol, isomalt, maltitol). They are formed via the catalytic hydrogenation of carbohydrates, during which, the aldehyde or ketone group is replaced by a hydroxyl group. Currently, there are eight (8) polyols approved by the US FDA, which include erythritol, hydrogenated starch hydrolysates, isomalt, lactitol, maltitol, mannitol, sorbitol, and xylitol [64,65]. Unlike the sugars discussed above, polyols lack a carbonyl group capable of reacting with amino acids, thereby resulting in the Maillard reaction. Polyols are reported to be extremely stable during heat and enzymatic and chemical degradation [66]. In recent times, there has been a burgeoning interest in incorporating polyols into various types of dosage forms such as liquid, oral preparations, lozenges, and tablets. Polyols are highly potent; are low in caloric content; have high nutritional value; and most importantly, are non-carcinogenic [10]. Several authors have reported the use of polyols such as mannitol, sorbitol, and xylitol in the preparation of SDs and their effect on the enhancement of the dissolution rate of poorly soluble drugs. In order to realize the potential of polyols to be used as carriers in the successful preparation of SDs, it is important to review the physicochemical properties associated with these compounds. A summary of the various polyols and their physicochemical properties is presented in Table 3.

#### 2.2.1. Mannitol-Based SDs

Mannitol, also known as D-mannitol, is a six-carbon sugar alcohol, widely recognized as a popular pharmaceutical excipient [72]. It stands as the predominant polyol employed in the preparation of SDs, with the primary focus being on drug solubility enhancement. More than 50% of documented studies have used it as the carrier, and one of the possible reasons could be its low-to-non-hygroscopic nature compared with other polyols [68]. Two primary techniques dominate the preparation of SDs involving mannitol, namely, the fusion and solvent evaporation methods. Notably, mannitol–drug SDs prepared through the fusion method often result in the formation of crystalline suspensions, as mannitol recrystallizes upon cooling from its molten state to room temperature. This can be attributed to its low *T_g_*, which is below room temperature at ≈11 °C [71]. This behaviour has been observed with various drug–mannitol SDs, such as clotrimazole [7], irbesartan [71], diacerein [73], carvedilol [74], and ketoprofen [75], to name only a few. This highlights mannitol’s limitations in forming ASDs and raises concerns about the thermodynamic stability of these SDs. To mitigate these challenges and enhance stability, research has explored the incorporation of antiplasticizers, such as hydrophilic surfactants like poloxamer 407 and hydrophilic polymers like PVP K30. These additives have been reported to effectively reduce the sugar component’s recrystallisation during ageing, leading to improved thermodynamic stability. In addition to improved stability, the ternary systems display higher dissolution rates because of a synergistic effect [76,77]. 

The enhancement of solubility and the dissolution rate can be attributed to two key factors. Firstly, this arises from the improved wetting of drug crystals owing to the attachment of mannitol particles to the surface [78], and secondly, this is the result of mannitol’s remarkable ability to form a strong hydrogen bond network within water [7,71]. The solubility enhancement of mannitol SDs is influenced by both the concentration and chosen preparation method. Several studies have found a positive correlation between an increase in mannitol concentration and maximum solubility, with the fusion method often yielding the highest solubility levels [73,79,80]. In general, both physical mixtures and SDs exhibit increased solubility and dissolution rates, with SDs demonstrating a notably greater improvement compared with the corresponding physical mixtures. Just as with sugar-based SDs, the solubility and dissolution enhancement primarily stem from the reduced particle size of the drugs and wettability, both facilitated by mannitol. Moreover, comparisons with other sugars have consistently shown that mannitol-based SDs outperform in terms of solubility, dissolution [7,37,40,44], and resistance to high temperatures [78]. 

Mannitol-based SDs prepared using the solvent evaporation method have been reported to enhance the solubility and dissolution rates of poorly water-soluble drugs (Table 3). This is attributable to reduced particle size, resulting in the formation of a solid solution, thus preventing drug particle agglomeration during dissolution and improving wettability. The characterization of SDs using techniques like DSC has revealed a decrease in the melting point [81] and a broadening or disappearance of the melting peak [82,83], which are indicative of the reduced particle size or amorphization of the drug. Along with the positive effect on solubility and the dissolution rate, mannitol-based SDs have been reported to enhance powder flow properties [84,85] and drug permeation efficacy [81]. It is worth noting that, while none of these studies have reported the formation of a completely amorphous system, they have often observed the drug becoming amorphous while mannitol remained in the crystalline state [44,81,86,87]. This phenomenon can be attributed to the evaporation of the solvent at high temperatures (50–70 °C), causing phase separation because of the increased molecular mobility of the mannitol. Alternative methods such as spray-drying, freeze-drying and spray–freeze-drying have been recommended to prevent phase separation [31]. 

Itraconazole [88], diazepam [89], and tadalafil [90] SDs with mannitol were prepared by employing spray-drying, which involved the use of hydro-alcoholic solvents to dissolve the drug and mannitol. While all conducted studies have consistently demonstrated a significant increase in the solubility of SDs, mannitol retains its crystalline structure [88,89]. This can be attributed to mannitol’s *T_g_*, which is below ambient temperature [89]. Interestingly, none of these studies have reported stability assessments. Thakur et al. [91] investigated the effect of mannitol’s surface characteristics (surface roughness, polar chemical environment, and porosity) on drug recrystallisation within SDs prepared via spray-drying. Their findings revealed that mannitol in the spray-dried product possessed higher roughness, a more polar chemical environment, and higher porosity, which facilitated the molecular re-orientation of amorphous fenofibrate, ultimately leading to recrystallisation. 

El-Maradny and Saab [90] used amino acids as co-formers to improve the aqueous solubility and the physical stability of SDs. The results indicated a significant increase in solubility and the dissolution rate for drug–amino acid–mannitol SDs compared with the SDs of binary mixtures. The inclusion of amino acids resulted in the formation of a co-amorphous system, wherein mannitol also underwent amorphization. Importantly, these resultant SDs were found to be stable for 6 months at 40 °C. The novel spray-drying technique NanoCrySP™ proposed by Thakur et al. [91] uses the nanocrystals of the drug to prepare a nanocrystalline solid dispersion (NSD). It will be interesting to explore the use of these findings in a wider spectrum of APIs and polyols, where ASDs can be generated using spray-drying in the presence of amino acids. 

Mannitol is widely used as a bulking agent in pharmaceutical formulations, particularly in freeze-dried injectables, because of its favourable physicochemical properties, such as non-hygroscopicity and the high eutectic temperature of mannitol–water systems, thus enabling primary drying at elevated temperatures [92]. In the realm of freeze-dried SDs, mannitol has been employed to produce both partial and complete ASDs with lovastatin [93] and acetazolamide [94], respectively. In stark contrast to sugar-based carriers, ASDs formed with mannitol do not exhibit solution-mediated phase transitions and show good stability for acetazolamide ASDs for 6 months at 40 ± 2 °C and 75% ± 5% RH. 

Apart from the aforementioned techniques, co-grinding with mannitol has been employed as an alternative technique to overcome the limitations of the fusion and solvent evaporation methods. This approach addresses issues, such as drug degradation, associated with the fusion technique—especially when there is a significant difference between the melting temperatures of the individual compounds—and toxicity risks linked to residual solvents in the case of solvent evaporation [95]. Valsartan–mannitol SDs were prepared using ball milling [96,97]. The outcomes of these studies indicated that the speed and time of milling are major factors in converting both components into an amorphous form, necessitating milling for over two hours. The presence of mannitol positively impacted the dissolution rates because of improved wettability in comparison with the milled neat drug caused by the self-aggregation of the drug particles. To overcome upscaling challenges associated with ball milling, Muehlenfeld et al. [95] introduced a continuous co-grinding process using an air-jet mill. They studied the solubility of micronized griseofulvin SDs with mannitol. The SDs formed in this manner were crystalline in nature, unlike the amorphous systems formed via ball milling. Both these techniques resulted in increased griseofulvin dissolution rates because of reduced crystallinity, improved wetting, and the homogeneous dispersion of the drug in the SDs. Interestingly, mannitol-based SDs prepared using all the aforementioned techniques did not show solution-mediated recrystallisation, which is noteworthy, this phenomenon is commonly seen among sugars. 

#### 2.2.2. Sorbitol-Based Solid Dispersions

Sorbitol, an isomer of maltitol [98], is another popular sugar alcohol with extensive applications in the pharmaceutical realm. It finds utility in the formulation of oral dosage forms serving as fillers, sweeteners, fast disintegrants, stabilizers, sugar substitutes in syrups, humectants in topical formulations, and isotonicity-adjusting agents in various parenteral formulations [99]. Similar to mannitol, studies have reported sorbitol–drug SDs prepared via the fusion and solvent evaporation methods as consistently resulting in the formation of crystalline suspensions. Interestingly, sorbitol showed better solubility and dissolution enhancement over mannitol [37] and other hydrophilic carriers such as polyvinylpyrrolidone (PVP K-25, PVP K-30), polyethylene glycols (PEG 4000, PEG 6000), and hydroxy propyl methyl cellulose (HPMC) [100,101]. Remarkably, akin to mannitol, no solution-mediated recrystallisation was observed in the studies reviewed. However, the hygroscopic nature of sorbitol, in contrast to mannitol [36] and all other polyols [68], may pose potential challenges concerning long-term stability. 

#### 2.2.3. Xylitol-Based Solid Dispersions

Xylitol, a pentahydroxy sugar alcohol that naturally occurs in various fruits and vegetables, stands out as one of the most extensively produced and employed sweeteners within the pharmaceutical industry [102]. Much like mannitol, xylitol is also one of the early polyols considered for the preparation of SDs aimed at achieving solubility enhancement. The pioneering work of Sirenius et al. in 1979 [103] marked its initial application carrier in the preparation of SDs containing p-aminobenzoates. These authors highlighted its suitability to be used as an SD carrier because of its low melting point and high thermal stability. While detailed molecular arrangements within these SDs are less documented, they were reported to achieve an up to 200-fold increase in solubility for SDs containing 5% butyl p-aminobenzoate. Solid glass dispersions of famotidine–xylitol were reported to form a two-component system, with xylitol maintaining its crystalline state and famotidine displaying a broad peak during DSC analysis, indicating reduced particle size. Unlike the sugar-based SDs mentioned earlier, a single-phase drug release pattern was observed with no recrystallisation occurring. Particularly noteworthy is the finding that a 1:40 ratio of drug to xylitol formed a eutectic system that yielded the most substantial aqueous solubility enhancement (31.7%) [104]. Similar results were reported in several studies, where xylitol-based solid dispersions formed a two-phase solid crystalline suspension [76,105,106,107]. 

Xylitol-based SDs are predominantly prepared using the fusion method and its modified counterpart, hot-melt extrusion (HME). In all cases, these dispersions enhance solubility and dissolution rates by improving the wettability of the drug particles. Studies have indicated that xylitol SDs show either a similar or superior solubility and dissolution behaviour compared with sorbitol [76] and mannitol [107], respectively. A key challenge highlighted in these studies is a lack of thermodynamic stability, attributable to crystal growth during storage and resulting in reduced dissolution rates [76,105,106]. Notably, xylitol is reported to possess a *T_g_* in the sub-zero temperature range (−19 °C to −24 °C) [69], causing it to become crystalline upon cooling to room temperature. This molecular mobility of drug particles leads to recrystallisation. However, SDs filled into hard gelatin capsules exhibited good stability over 3 months when stored under accelerated conditions [107]. Collectively, the findings of these studies suggest that xylitol is a promising carrier for improving the solubility and dissolution of poorly soluble drugs. 

#### 2.2.4. Erythritol-Based Solid Dispersions

Erythritol, another naturally occurring four-carbon polyol, is commonly used as a bulk sweetener in both food and pharmaceutical applications, owing to attributes such as its negligible caloric value, excellent heat stability, and low water activity, rendering it non-hygroscopic [68,108,109]. However, its low aqueous solubility and propensity for rapid crystallisation [110], which could be attributed to its low *T_g_* of approximately −45 °C [69], could be some of the reasons why no studies have reported SDs prepared with the heat–cool and freeze-drying methods. Nishimoto et al. [111] reported using erythritol in the preparation of ternary ASDs comprising griseofulvin and hypromellose phthalate through the hot-melt extrusion method. The addition of erythritol imparted hydrophilicity and reduced the viscoelasticity of the system, resulting in enhanced solubility and stability by maintaining the amorphous state of the hypromellose phthalate. Additionally, erythritol is reported to have good solubility in organic solvents such as ethanol and methanol [112], and it will be interesting to explore its use as a carrier for SDs prepared using the solvent evaporation technique. 

#### 2.2.5. Isomalt-Based Solid Dispersions

Isomalt, comprising an equimolar mixture of the two disaccharide alcohols (alpha-D-glucopyranosyl-[1-6]-D-sorbitol (GPS) and alpha-Dglucopyranosyl-[1-6]-D-mannitol (GPM) [113]), shares similarities with erythritol, such as its very low hygroscopicity. What makes it interesting in comparison with other polyols is its ability to remain glassy after re-solidification. This is attributable to its high *T_g_* of 61.5 °C, surpassing all other polyols [70], consequently making it a suitable carrier for the preparation of SDs using the fusion method. Despite this advantage, no studies could be found in the literature for isomalt-based SDs prepared with the fusion technique. 

The scant existing studies on isomalt-based SDs have exclusively employed the spray-drying technique with ethanol as the solvent. SDs prepared with isomalt result in glassy SDs, where the drug is converted into an amorphous state and the isomalt particle size is reduced substantially. While the solubility and dissolution rate improve for the binary SDs of PVP and isomalt, the ternary SDs of the drug, PVP and isomalt, perform better. Comparative studies of PVP and isomalt as carriers have indicated the superior performance of isomalt in regard to yield [114]. Stability studies conducted on 10% SDs of celecoxib [114] and indomethacin [115] produced varying outcomes. Celecoxib reverted to a crystalline form after 1 month of storage, whereas indomethacin remained in an amorphous state. This difference in stability could be explained based on the glass-forming capabilities of the studied drugs, where celecoxib exhibits moderate glass stability [116] and indomethacin possesses good glass-forming ability [117]. It has also been suggested that the high number of hydroxyl groups in isomalt fosters better interaction with indomethacin and consequently causes better physical stability. One major problem observed with these studies is the loading capacity of the drug, which is between 10 and 30%, making the final dosage form bulky. 

#### 2.2.6. Maltitol-Based Solid Dispersions

Maltitol is another interesting polyol that has physical properties similar to isomalt. It is also a disaccharide polyol (4-O-α-D-glucopyranosyl-D-glucitol) composed of glucose and sorbitol in equal parts [65]. Its noteworthy physical properties include its high water solubility, similar to maltitol [118]; a relatively high *T_g_* of 49.5 °C; low hygroscopicity; and a significant number of hydroxyl groups that could foster enhanced drug interaction. Maltitol can also withstand high temperatures, as it is reported to degrade at 274 °C [119]. Analogous to isomalt, there appears to be an absence of research documenting its application in the preparation of SDs via the fusion technique. However, research led by Koreyoshi Imamura and his research team has explored its use as a carrier in the preparation of ASDs using the freeze-drying technique [54,56,57]. Although these studies did not report great success in creating maltitol SDs over other sugar carriers (viz. α-maltose, palatinose, and trehalose), its potential in forming ASDs should not be discounted. Given its favourable physicochemical properties, this merits investigation into the preparation of SDs using alternative techniques such as fusion and HME. 

Observations made from the studies reviewed confirm that polyols are gaining popularity as sugar substitutes. However, it is important to note that the selection of the appropriate polyol as a carrier for SDs depends on the specific physicochemical characteristics (see Table 3 and Table 4), such as *T_g_* and crystallisation kinetics, which determine the selection of a suitable technique to be used. Polyols such as xylitol, erythritol, isomalt, and maltitol remain relatively underexplored despite their favourable physicochemical properties, warranting further investigation and consideration in future studies.

## 3. Co-Amorphous and Co-Crystalline Systems

Co-amorphous and co-crystalline systems are an emerging formulation approach involving combining the drug with hydrophilic, small-molecular-weight compounds such as amino acids, carboxylic acids, and sugars in the preparation of drug–excipient systems in order to improve drug solubility and bioavailability. Studies have indicated that these drug–excipient systems yield improved dissolution rates and stability compared with their respective crystalline or amorphous counterparts. These improvements are facilitated through intermolecular interactions such as hydrogen bonding and π–π interactions [120,121]. Co-amorphous systems stabilize the amorphous forms through strong and specific molecular interactions between the drug and partner molecule and by increasing the glass transition temperature. In this system, amorphous forms are quite stable, as they need a stronger effort to go back to their crystalline state, in that they first have to break the molecular interactions and then rearrange their molecules to form crystalline nuclei [122]. Also, the availability of a wide range of suitable co-formers, compared with counterions for salt formation, makes this approach attractive [123,124]. By selecting an appropriate co-former, co-crystals offer tailormade solutions to various solid-state problems, such as physicochemical stability, including photostability, hygroscopicity, permeability, compressibility, flow properties, solubility, bioavailability, pharmacokinetic properties, etc., without altering the molecular structure and pharmacological properties [123,125].

It is worthwhile to note that no studies have explored the formation of co-amorphous systems using sugars or polyols; only one study has surfaced pertaining to co-crystals with polyols. Arafa et al. [16] reported xylitol as a potential co-former in the preparation of felodipine co-crystals. The authors reported successful co-crystal formation with a 1:2 ratio of felodipine to xylitol using a wet co-grinding method. The characterization of the co-crystals with DSC showed two characteristic melting peaks in the thermogram, corresponding to both the carrier and the drug. Typically, co-crystals are single-phase systems [126] that are characterised by the presence of a single endothermic peak between the melting temperatures of the individual compounds [8,127]. The lack of single-crystal XRD data also makes it difficult to confirm the formation of co-crystals with xylitol. With the supramolecular synthon approach, polyols have the potential to form co-crystals. The propensity of sugars to engage in a multitude of hydrogen bonds has established their significance in supramolecular chemistry. A heterosynthon between the hydroxyl groups of polyols and drugs consisting of functional groups such as ether could conceivably result in the formation of a co-crystal. However, this prospect warrants further research to definitively establish it. 

## 4. Natural Deep Eutectic Solvents

In the quest to address the inherent toxicity of organic solvents used in the food, chemical, and pharmaceutical industries, alternative green and biodegradable solvents such as ionic liquids (ILs), deep eutectic solvents (DESs), and natural deep eutectic solvents (NADESs) have emerged. While the “greenness” of ILs is often questioned, DESs have been extensively studied for their pharmaceutical applications to improve the solubility and bioavailability of poorly soluble drugs. However, their safety is also questionable, as not much data from in vivo and long-term stability are available [128,129]. NADESs are a mixture of two or more components from natural sources, including sugars, sugar alcohols, poly alcohols, amino acids, and organic acids [129,130]. NADESs are considered superior to DESs because of their unique physicochemical properties, such as low volatility, an ability to remain in the liquid state in sub-zero temperatures, biodegradability, and ease of preparation [131]. Despite the advantages reported, its applications in pharmaceutical research have not gained full momentum yet. 

In two different studies by Jeliński et al. [131,132], various sugars (fructose, glucose, maltose, sucrose) and polyols (xylitol, sorbitol) were investigated for the solubility enhancement of sulphanilamide and curcumin, respectively. NADESs were prepared with choline chloride as a primary constituent, and one of the sugars or polyols was mixed with it in different molar ratios. All the resultant NADESs showed a two-fold increase in solubility for sulphanilamide and a significant increase in curcumin, with the lowest being 1000-fold. Maugeri and De Marı’a [14] reported the neutral pH for sugar-based deep eutectic solvents, which is of considerable importance in pharmaceutical applications, mainly in terms of stability. In addition to these studies, Dai et al. [133] reported the possibility of preparing NADESs with different combinations of sugars and polyols. Though there is limited literature available on the pharmaceutical applications of NADESs, their applications as extraction media are well documented [129,134]. The fundamental knowledge obtained from these studies can be extrapolated to address the solubility challenges of various BCS Class II and IV drugs. 

## 5. Conclusions and Future Perspectives

There is growing interest in utilizing low-molecular-weight, water-soluble carriers for enhancing the solubility and dissolution rates of poorly soluble drugs. Initially, sugars were the preferred carriers in the formulation of first-generation SDs given their low molecular weight and hydrophilic nature. However, their application is met with several challenges, primarily their low thermal stability, limited miscibility, and hygroscopic nature. As the majority of the SDs formed using sugars result in crystalline suspensions, the drug release from these formulations has been found to be much slower compared with amorphous forms. The use of polyols has effectively addressed these challenges and has proved to be a better choice of carriers over sugars. The effectiveness of these carriers in enhancing the solubility of drugs is dependent on a number of factors, including physicochemical properties such as the melting point, *T_g_*, the miscibility of drugs in the carrier, etc. The preparation method also plays a significant role, with the fusion technique showing more success over solvent evaporation, mainly because of the limited solubility of the sugars and polyols in organic solvents. 

The use of ultrasonication and freeze-drying have shown potential in improving miscibility and the formation of ASDs with both sugars and polyols; however, these ASDs have been found to be unstable. Strategies such as the preparation of ternary solid dispersions by including amino acids, synthetic hydrophilic polymers, or poloxamers have proven to be highly successful in obtaining stable ASDs. Second-generation SDs, which can achieve this without the inclusion of sugars or polyols, come with unique challenges, such as requiring high concentrations of polymers, which can lead to viscosity increases during dissolution and imparting their characteristic taste in the case of amino acids. Considering that sugars and polyols are primarily used in the pharmaceutical industry for their taste-masking properties and solubilizing capacity, they can be the preferred choice of carriers in the preparation of ternary solid dispersions, which is the current direction of research. Novel techniques such as NanoCrySP™ used to prepare nanocrystalline solid dispersions with polyols should also be explored, as they also show very promising results. 

In this review, it is evident that polyols such as xylitol, erythritol, isomalt, and maltitol are relatively underexplored. The preliminary observations suggest that they have potential applications in formulating stable SDs, and the good glass-forming abilities of isomalt and maltitol are of particular interest for preparing ASDs. In addition to this, preparing drug–excipient-based co-amorphous and co-crystalline systems has seen limited exploration, with only a few low-molecular-weight excipients being explored. Given the potential of these systems to improve the dissolution characteristics of poorly soluble drugs, the proven ability of some low-molecular-weight artificial sugars, such as saccharin and sucralose, provides the background for exploring low-molecular-weight polyols as potential excipients. Moreover, the use of natural sugars and polyols in the formulation of NADES-based drug delivery systems presents great research opportunities. 

## Figures and Tables

**Table 1:** Summary of solid dispersion techniques and their advantages and limitations.

Method	Advantages	Limitations	Ref.
Fusion	Simple and cost-effective. Suitable for a wide range of compounds.	Thermal degradation of the carrier, API. Difficult to control particle size and distribution.	[29,30]
HME	Uniform distribution of the drug particles. Short heating time. Continuous and scalable process.	Heat-sensitive drugs.	[19,29,30,31]
KM	Simple and cost-effective.	Difficulty with removing solvents. Difficult to control particle size and distribution.	[25]
SE	Better control of particle size and distribution. Suitable for heat-sensitive compounds.	Difficulty with removing solvents. Common solvent system required. Phase separation due to differences in polarity.	[19,27,29]
Co-precipitation	Simple and cost-effective. Suitable for compounds with similar solubility profiles.	Limited to certain drug–excipient combinations. Plasticization effect of water absorbed.	[25]
SCF	Excellent drug and carrier distribution. Suitable for heat-sensitive compounds.	Limited solubility of APIs and carriers in CO_2_.	[19,24]
SD	High drug loading and excellent control over particle size. Excellent flow properties. Suitable for thermally labile compounds. Continuous and scalable process.	Requires the use of organic solvents, which must be removed. Equipment can be expensive. Common solvent system required to prevent phase separation.	[25,32]
FD	Suitable for thermally labile compounds. Can produce highly porous structures.	Time-consuming process. Limited drug loading due to the porous structure.	[25,31]
ES	Excellent control over particle size and morphology.	Requires specialized equipment. Limited drug loading capacity. Batch-to-batch variability. Limited high molecular weight carriers.	[25,31]
Milling	Simple and scalable process.	Heat-sensitive drugs. Sticks to the walls of the milling chamber.	[33]

HME = hot-melt extrusion; KM = kneading method; SE = solvent evaporation; SCF = super critical fluid; SD = spray-drying; FD = freeze-drying; ES = electrospinning.

**Table 3:** Polyols and their physicochemical properties

Polyol	Structure	MW (g/mol)	HBD/HBA	MP (°C)	*T_g_* (°C)	Aq.S (% at 20 °C)	Ref.
Erythritol	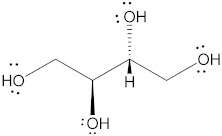	122.12	4/4	118–126	−45.0–−42.50	37	[67,68,69]
Isomalt	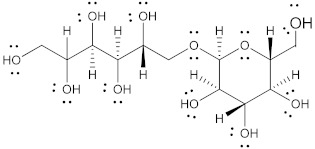	344.31	9/11	98 and 155	61.5	25	[67,68,70]
Mannitol	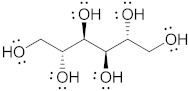	182.17	6/6	164–176	10.7	20	[68,70,71]
Maltitol	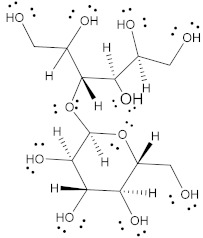	344.31	9/11	148–151	43.1–49.5	60	[67,68,70]
Sorbitol	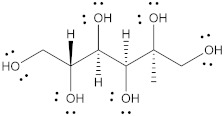	182.17	6/6	95–97	−9.20–−6.0	73	[67,68,69]
Xylitol	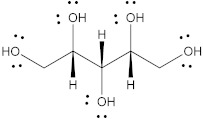	152.15	5/5	92.7	−24.10	63	[68,69]

**Table 4:** Summary of SDs reported in the literature obtained by using various polyols as carriers.

Polyol	Method	Drug: Carrier (*w*/*w*)	Drug	Solubility	Dissolution	Remarks	Ref.
Mannitol	Fusion	1:3, 1:1, and 3:1	Clotrimazole	Significant increase at 1:13 ratio.	Increase with increase in mannitol concentration.	Partial dispersion of drug at molecular level.	[7]
	Fusion	1:1	Sulfamethoxazole	No significant change with increase in carrier concentration.	A 100% drug release in 5 min.	Partial dispersion of drug at molecular level.	[37]
	Fusion	4:1, 2:1, 1:1, and 1:4	Ibuprofen	N/A	A 100% drug release within 60 min at 1:4 ratio.	SDs of hydrophilic polymers released 100% within 15 min	[40]
	SE	1:1 and 1:5	Etoricoxib	1.4 to 1.8-fold at 1:5 ratio.	N/A	Intermolecular hydrogen bonding between S=O group of etoricoxib and O–H group of mannitol.	[44]
	Quench Cooling	N/A	Irbesartan	Increased solubility reported.	Lower dissolution compared with pure drug due to formation of hard plug by SDs.	Mannitol does not affect the pH of the dissolution medium.	[71]
	Fusion and SE	1:1, 1:3, and 1:5	Celecoxib	Increase of 1.3- and 1.2-fold at a 1:5 ratio for fusion and SE, respectively.	Increases of 9- and 6-fold in drug release by 60 min for fusion and SE, respectively.	No intermolecular interactions formed.	[73]
	Fusion	1:1, 1:2, 1:3, and 1:5	Carvedilol	N/A	Close to 100% drug release by 30 min for SDs as compared to 53% for pure drug.	No intermolecular interactions formed.	[74]
	Fusion and Kneading	1:1, 1:3, and 1:5	Ketoprofen	Increase in solubility with an increase in carrier concentration.	Six-fold increase in comparison with the pure drug by 30 min.	No intermolecular interactions formed.	[75]
	Fusion	1:1 and 1:9	Nifedipine	N/A	SDs at 1:9 ratio showed better dissolution rate.	No intermolecular interactions formed.	[78]
	Fusion, Kneading and SE	1:1, 1:2, 1:3, 1:4, and 1:5	Azithromycin	Solubility in the order of fusion > SE > kneading	N/A	Solubility decreased at 1:4 ratio. Possible reason could be increased viscosity at high sugar concentrations.	[79]
	Fusion and SE	1:1, 1:3, and 1:5	Diacerein	Increase of 27- and 26-fold at a 1:5 ratio for fusion and SE, respectively.	A 100% and 87% drug release by 60 min for fusion and SE, respectively, in comparison with 53% for pure drug.	Possible hydrogen bonding between the carrier and drug with fusion method.	[80]
	SE and SD	1:1, 1:2, 1:4, 1:6,1:8, and 1:10	Olanzapine	N/A	Increase in dissolution rate with increase in carrier concentration.	Increased dissolution is associated with the decreased crystallinity of the drug.	[81]
	SE	1:2 and 1:3	Aceclofenac	N/A	Mannitol > dextrose > HPMC > PVA	None	[84]
		1:1, 1:2, and 1:4	Carvedilol	PEG > lactose > mannitol	PEG > lactose > mannitol	Increase in solubility and dissolution with increase in carrier concentration.	[85]
	SE	1:1, 1:2, 1:4, 1:6,1:8, and 1:10	Clozapine	N/A	Increase in dissolution rate with increase in carrier concentration and no significant change beyond 1:2.	Increased dissolution is associated with the decreased crystallinity of the drug.	[87]
	SD	N/A	Diazepam	N/A	Dissolution rate increased with increasing water/organic solvent ratio.	Crystallinity of diazepam decreased when the water/organic solvent ratio increased.	[89]
	SD	1:1, 1:2, and 1:4	Tadalafil	3 to 6-fold increases	Three- to ten-fold increase.	ASD formed were stable for 6 months at 40 °C.	[90]
	FD	Various ratios (between 1:1 and 1:2)	Lovastatin	5–6-fold increase	Tablets of SDs mixed with hydrophilic polymers provided 1.4-fold increase.	SDs are amorphous in nature with particle sizes between 100 to 1000 nm.	[93]
	FD	1:0.5, 1:1, and 1:2	Acetazolamide	5 to 6-fold increase at 1:1 ratio	Superior performance by ASD at a 1:1 ratio with >90% drug release by 60 min.	ASD with 1:1 ratio was stable for 6 months with no significant change in the crystallinity.	[94]
	Milling	1:1, 1:3, and 1:9	Griseofulvin	N/A	Significant increase in % drug release for all the SDs.	No significant difference in % drug release between physical mixtures and SDs.	[95]
	Milling	1:1 and 1:3	Valsartan	N/A	Tablets of SDs showed >90% drug release in 30 min in comparison with <20% pure drug.	No significant difference in % drug release between physical mixtures and SDs.	[96]
	Milling	1:1, 1:3, and 1:5	Valsartan	N/A	SDs showed >90% drug release in 60 min in comparison with <40% pure drug.	No significant difference in % drug release between SDs with various ratios of mannitol.	[97]
Sorbitol	SE	1:1, 1:3, and 1:5	Diacerein	N/A	Increase in dissolution rate over pure drug and physical mixtures in the order of sorbitol > PEG400 > PVPk25.	Tablets prepared with SDs were stable at 30 °C/75% RH and 40 °C/75% RH for 12 weeks.	[100]
	Fusion and SE	1:1, 1:2, 1:3, and 1:4	Ritonavir	2000-fold increase in solubility at a 1:4 ratio	Two-fold and four-fold increases for SDs prepared with SE and fusion methods, respectively.	Hydrogen bond formation through interaction with amide and carbonyl groups of drug and hydroxyl groups of sorbitol.	[101]
Xylitol	Fusion	1:9	Etoricoxib	N/A	Two-fold increase	Ternary and quaternary systems performed better than the binary systems.	[76]
	Fusion	5%	p-Aminobenzoates	N/A	A 200-fold increase.	N/A	[103]
	Fusion	1:20 and 1:40	Famotidine	A 32% increase in solubility over pure drug for 1:40 SD.	A 100% drug release in less than a minute for 1:20 SD, compared to 50% for pure drug.	Eutectic mixture formed at 1:40.	[104]
	HME	1:1 and 1:4	Efavirenz	An 81-fold increase for 1:4 SD.	A 4.1-fold increase in dissolution rate.	Reduced dissolution rate during storage during storage.	[105]
	HME	1:4 and 2:3	Carbamazepine	Aqueous solubility is increased by 50-fold.	A 100% drug release in 15 min in comparison with 40% for the pure drug.	Stable for 3 months under accelerated conditions.	[107]
Erythritol	HME	Various	Griseofulvin	A 10-fold increase in solubility.	N/A	Substantial reduction in melting point of the drug, resulting in reduced processing temperature.	[111]
Isomalt	SD	Various	Celecoxib	A 5-fold increase in solubility.	A 94% drug release after 5 min for ternary systems in comparison with 31% for the pure drug.	Binary systems of drug-to-isomalt ratio showed recrystallisation after 1 week. Ternary systems with PVP were stable.	[114]
	SD	2%, 10%, and 30%	Indomethacin	N/A	Rate of dissolution decreased with increase in carrier concertation.	Isomalt SDs showed better physical stability compared with PVP SDs.	[115]

SE = solvent evaporation; SD = spray-drying; FD = freeze-drying; HME = hot-melt extrusion.

## Data Availability

The data collected in this study are presented and available in this article.
